# Application of a Posttreatment to Improve the Viability and Antifungal Activity of *Trichoderma asperellum* Biomass Obtained in a Bioreactor during Submerged Cultivation

**DOI:** 10.3390/biology11111610

**Published:** 2022-11-03

**Authors:** Maris Senkovs, Marija Tereze Dzierkale, Alina Rimkus, Oskars Grigs, Vizma Nikolajeva

**Affiliations:** 1 Faculty of Biology, University of Latvia, Jelgavas Street 1, LV-1004 Riga, Latvia; 2 Bioefekts Ltd., Livzemes Street 30, LV-2169 Salaspils, Latvia; 3 Latvian State Institute of Wood Chemistry, Dzerbenes Street 27, LV-1006 Riga, Latvia

**Keywords:** *Trichoderma*, fungal biomass, *Fusarium*, antifungal activity, viability, submerged fermentation

## Abstract

**Simple Summary:**

*Trichoderma* spp. are common soil microorganisms that play an important role in limiting phytopathogenic microorganisms, improving plant growth and degrading plant biomass. Often the determining factors affecting the growth and maintenance of viability of *Trichoderma* spp. are the composition and condition of the growth medium. This study provides information on post-treatment procedures that would improve the viability of *T. asperellum* biomass and the antifungal activity obtained from submerged cultivation in a bioreactor. The aim of the study was to determine the viability of fungal biomass and competitiveness against a phytopathogen after treatment with hydrochloric acid, copper (II) sulphate and starch, alone or in combination.

**Abstract:**

*T. asperellum* MSCL 309 was used in the study. *T. asperellum* was grown in the stirred bioreactor under submerged cultivation. The resulting biomass was filtered to obtain a thick biomass. The viability and antifungal activity of *T. asperellum* biomass samples were determined simultaneously by studying the colonization of the malt extract agar medium surface and its competitiveness with the plant pathogenic fungus *Fusarium graminearum* using in vitro dual culture experiments. Treatment with starch, alone or in combination with copper (II) sulphate and/or hydrochloric acid did not significantly affect fungal viability compared to control. An important factor in maintaining viability was the addition of hydrochloric acid, which significantly increased the storage life of biomass. In all post-treatments, *F. graminearum* was overgrown with *T. asperellum* in seven days, and accordingly, the larger the area occupied by *T. asperellum*, the smaller the area of *F. graminearum* colonization. Viability and antifungal activity of *T. asperellum* persisted throughout the experiment, at least for eight weeks. All the post-treatment methods we studied improved the viability and antifungal activity of *Trichoderma*, at least in terms of the area of the colonized surface. For the development of long-term viable and active *T. asperellum* preparations, we recommend the use of acidification of *T. asperellum* biomass obtained by submerged fermentation.

## 1. Introduction

*Trichoderma* species are soil fungi whose most important functions in the ecosystem are to degrade plant biomass, but they also limit phytopathogenic microorganisms and improve plant growth [1,2]. For more than 70 years, *Trichoderma* spp. have been used in crop production as biocontrol agents, biofertilizers and biostimulants [3]. The genus *Trichoderma* also plays an important role in the bioremediation of contaminated soils [4,5]. As these fungi are widespread in the soil, such biopreparations do not create an imbalance in the ecosystem. *Trichoderma* spp. are highly adaptive to the environment, and their growth rate is generally higher than that of plant pathogens. *Trichoderma* spp. can compete with adjacent pathogenic microorganisms for a zone of existence or nutrients, thereby inhibiting the growth of pathogens [1]. The main mechanisms of biocontrol of *Trichoderma* spp. are antibiosis, competition and mycoparasitism [6]. *Trichoderma* spp. have shown the ability to compete, for example, with plant pathogenic and mycotoxigenic fungi of the genus *Fusarium* in vitro in dual confrontation assays [7]. In plant experiments, they reduce the incidence and severity of diseases, mainly as preventive agents and by secreting phytohormones and cell membrane degrading enzymes [8]. Secondary metabolites synthesized by *Trichoderma* play an important role both in chemical protection against plant pathogens and in communication with other organisms [9].

The composition of the medium and the conditions under which fungi of the genus *Trichoderma* are cultivated have a significant effect on their growth and viability. There are two general approaches regarding *Trichoderma* fermentation: submerged (liquid) fermentation (SmF) and solid-state fermentation (SSF) [10]. In comparison to SmF, SSF has lower capital costs and higher productivity, and the produced propagules are more stable, simpler and have cheaper downstream processing, lower wastewater discharge and reduced energy requirements. However, SmF processes are much less labor intensive and easier to control and automate [11]. *Trichoderma* growth, conidiation and the production of antimicrobial compounds are highly affected by the oxygen transfer rate controlled by aeration and agitation [12]. The morphology of the fungi is also influenced by the composition of the nutrient components in the medium, pH, temperature, etc. [13], and the efficacy of biocontrol agents to suppress plant pathogens varies depending on the nutrient composition of the medium [14].

*Trichoderma* species reproduce asexually by producing three major types of propagules (mycelia, conidia, and chlamydospores) [12,15] that possess distinct physiological characteristics in terms of production, stability, and biocontrol activity. *Trichoderma* fungi usually form branched hyphae and chlamydospores under submerged fermentation, but conidia are rarely formed [12,16]. In nature, many species also form ascospores in perithecia [17]. In practice, chlamydosporal preparations are used in some cases [18], but most commercial formulations use aerial conidia [19]. Several soluble and volatile secondary metabolites—peptaibols, polyketides, pyrones, terpenes, etc.—are synthesized by the fungal mycelium just during conidiation [20]. According to available information, we fully agree with [21] that no reports on yields, fermentation time, production costs of liquid fermentation, and comparison with aerial conidia in terms of bioefficacy are available. Formulation studies have focused on stabilization processes for *Trichoderma* biomass, conidia, and chlamydospores [22]. Despite these attempts, low yields, long fermentation times, and poor storage stability have hampered the use of SmF.

Spores produced by the aerial mycelium of *Trichoderma* show both higher resistance and longer viability after storage than those produced in a liquid medium [23]. As noted in [16], conidia produced by SSF survive longer than chlamydospores and exhibit almost equal bioefficacy in reducing root rot incidence compared to chlamydospores produced under SmF. As conidia are formed predominantly in aerated conditions, this has led to a two-step production procedure: SmF application for mycelium and SSF application for sporulation [24].

The posttreatment of biomass obtained during SmF can be considered the first step in the two-step production procedure. The posttreatment possibilities have been studied in experiments with the genus *Trichoderma* [7] by adding substances to the biomass of *T. asperellum* that reduce metabolic activity and/or increase nutrient uptake. Acidification with hydrochloric acid inhibits the rate of fungal metabolism; low concentrations of copper sulphate act as a metabolic inhibitor, while starch serves as a source of nutrients to prolong shelf life. According to [7], biomass in Petri plates retains viability and antifungal activity for at least 6 months when stored at room temperature. The importance of oxygen availability in maintaining fungal viability should also be considered. It should also be noted that the environmental factors during fermentation influence both the antagonistic properties and duration of cell viability.

The present study aimed to assess the viability and antifungal activity of *T. asperellum* MSCL 309 biomass obtained in submerged fermentation and its preservation after treatment of biomass with hydrochloric acid, copper sulphate, starch, and their combinations.

## 2. Materials and Methods

### 2.1. Cultivation of Microorganisms

*Trichoderma asperellum* MSCL 309 was isolated from a temperate climate region (Latvia) and identified by amplification of the rRNA gene region with specific primers [25].

*T. asperellum* MSCL 309 was used in this study as a model fungus and *Fusarium graminearum* MSCL 435 as the model plant pathogen. Both fungi were grown on malt extract agar (MEA, Biolife, Milan, Italy) [26] in Petri plates at 20 ± 2 °C for 7 days.

To prepare the inoculum, *T. asperellum* was grown statically in malt extract broth in flasks at 28 °C for 57 h, followed by shaking at 150 rpm for 8 h. *T. asperellum* submerged cultivations were performed in a 15 L stirred-tank bioreactor (EDF-15.1, Biotehniskais centrs, Riga, Latvia) with one standard Rushton turbine (bottom location) and two propeller type turbines (middle located for flow up; above located for flow down). The medium used contained sugar (20 g/L) and yeast extract (3 g/L). 11.4 l of medium and 600 mL of inoculum were added to the bioreactor. The medium was sterilized for 30 min at 1.1 atm and 121 °C. Cultivation set points were a temperature of 28 °C, pH of 6.5 ± 0.2, and dissolved oxygen concentration of 30 ± 5%. Agitation limits ranged from 200 rpm up to 750 rpm, and the aeration rate was 1.67 standard liters per minute. The duration of cultivation was 65 h.

### 2.2. Posttreatment of T. asperellum Biomass

The obtained biomass was filtered through three layers of gauze to obtain a thick biomass with a moisture content of 88.2%. Moisture content was determined by weighing 10 g of biomass and heating in an AGS 120/T250 moisture analyzer (Axis, Gdańsk, Poland) at 85 °C for 25 min. The biomass was weighed into 12 sterile Petri plates (with three repetitions) 50 mm in diameter, weighing 5 g of biomass on each plate. This biomass was treated in several ways (Table 1) by modifying the [7] method:

(1) HCl (Stanchem, Warszawa, Poland), adding 200 µL of 1 M HCl to achieve a pH of 4;
(2) CuSO_4_×5H_2_O solution (Sigma-Aldrich, St. Louis, MO, USA) at 2 mg/mL, adding 50 µL to 20 µg/mL (or 100 µg/5 g of biomass) treatment or 150 µL to 60 µg/mL (or 300 µg/5 g of biomass) treatment; and
(3) organic potato starch (Aloja Starkelsen Ltd., Ungurpils, Alojas pagasts, Latvia), 500 mg, thoroughly mixed with biomass.

A total of 350 µL of sterile water was added for the control (treatment No. 1).

Samples were stored at 22 ± 1 °C and humidity was in the range of 55–65%. Each week, part of each sample was watered evenly with 4 mL of sterile distilled water. The viability and antifungal activity of *T. asperellum* were determined immediately after mixing the biomass with the substances, after 1 week, 2 weeks, 4 weeks, 6 weeks and 8 weeks.

### 2.3. Determination of Viability, Antifungal Activity and Micromorphology of T. asperellum

The viability and the antifungal activity of *T. asperellum* biomass samples were determined simultaneously by studying the colonization of the agar medium surface and its competitiveness with the plant pathogenic fungus *F. graminearum* [27]. Twelve Petri plates with MEA medium were prepared. Autoclaved sterilized filter paper discs with a diameter of 0.4 cm were uniformly moistened with a 1% suspension of *T. asperellum* prepared by mixing 0.1 g of *T. asperellum* biomass with 10 mL of sterile water. Biomass was collected and prepared in a laminar flow cabinet. A *T. asperellum* filter paper was placed on MEA medium at a distance of 4 cm from a 0.4 cm diameter *F. graminearum* agar plug cut from a previously prepared plate with seven-day-old culture. Petri plates with both cultures were stored for 7 days at room temperature 22 ± 1 °C. Viability was measured on the third day from the area of the colonized surface of *T. asperellum* and expressed as a percentage of the total surface area of the plate. Antifungal activity was determined on day seven, from the colonized surface area of *F. graminearum*. Fungal micromorphology was examined using a Leica DM 2000 microscope under 200 and 400× magnification, and images were recorded digitally with a Leica DFC 420 camera. ImageJ software 167 version 1.53e (National Institutes of Health, Washington, DC, USA) was used for image processing.

### 2.4. Statistical Analysis

Each experiment was performed in triplicate. The data were analyzed using the computer program RStudio, version 1.4.1103 (R Foundation for Statistical Computing, Vienna, Austria). For statistics, analysis of variance (ANOVA test) and Duncan’s new multiple range test (MRT) were used. Means were compared using the significance level *p* < 0.05.

## 3. Results

### 3.1. Viability of T. asperellum

Submerged fermentation yielded 11 L of final product, resulting in 585.2 g of wet biomass and a wet biomass yield of 53.2 g/L. Biomass posttreatments were performed during the study, and the effects of several treatments on the duration of viability of the obtained *T. asperellum* biomass were compared at 5 different times. Immediately after biomass treatment, *T. asperellum* colonized 30.3 ± 1.4% of the total area of the Petri plates over three days (Table 2). No significant differences were observed between the colonized surface from the differently treated samples (*p* > 0.05).

The colonized surfaces of *T. asperellum* stored for one week showed significant differences between treatments. On one side, the smallest areas were observed in treatments No. 8 and No. 12, being 17.9% of the total area of the plate. On the other side, the highest values were observed in treatments No. 9 and No. 10, being 26.2–26.3%. After a week starch + acid affected colonization capacity and starch + Cu (on both concentrations) promoted colonization. Significant reductions in area % (*p* < 0.05) were observed for treatments No. 2, No. 4 and No. 12.

There were significant differences (*p* < 0.05) between the results from weeks one and two for all the treatments. For *T. asperellum* biomass stored for two weeks, the smallest area of the colonized surface was observed in treatment No. 8, where it was 28.5% of the plate area, and the highest values were observed in treatments No. 10, No. 11 and No. 12, where they were 41.1%, 40.0% and 40.2% of the plate area, respectively.

Samples of *T. asperellum* stored for four weeks colonized an even larger surface area than those stored for two weeks, i.e., 68.1 ± 5.4% of the total plate area. A significant difference (*p* < 0.05) between the two- and four-week results was observed for all treatments. After two weeks starch + acid affected colonization capacity and, starch + Cu (60) and starch + acid + Cu at both concentrations promoted colonization.

Significant changes (*p* < 0.05) between the fourth and sixth weeks were observed only for treatments No. 2, No. 3 and No. 10 and were due to an increase in colonized area. Samples of *T. asperellum* stored for six weeks were able to colonize 69.4% and 67.6% of treatments No. 1 and No. 7 and 83.0% of treatment No. 2, respectively. A significant difference (*p* < 0.05) between the six- and eight-week results was observed for treatment Nos. 1–5, No. 9, and No. 11.

Samples stored for eight weeks colonized from 52.2% of the surface for treatment No. 1 up to 71.5% of the surface for treatment No. 9. Overall, in the eighth week, Cu (60) and starch, as well as acid + Cu (60) and starch promoted the highest percentage of colonization compared to other treatments, but these data were not significantly different from treatments No. 2, No. 3, and No. 6 (*p* > 0.05). Colonization percentage in samples with treatments No. 1, No. 4, No. 5, No. 7, No. 8, No. 9. and No. 11 differed significantly from samples No. 2, No. 3, No. 6, No. 10, and No. 12 (*p* < 0.05), where surface colonization with *T. asperellum* was the highest.

### 3.2. Morphological Features and Antifungal Activity of T. asperellum

**Figure 1:**
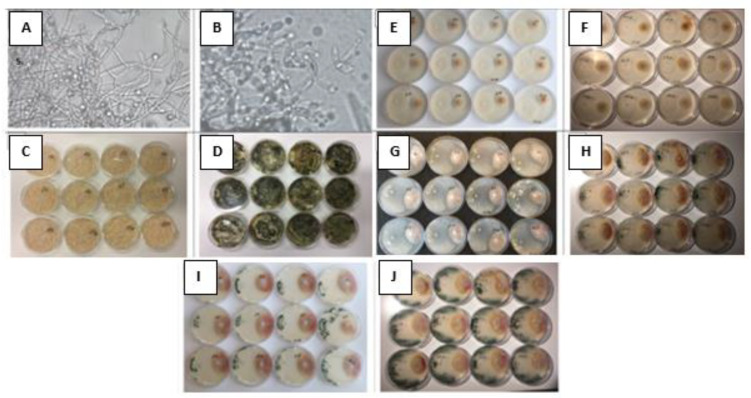
Different treated biomasses of *T. asperellum* and antagonistic action against *F. graminearum* in dual culture assays depending on the storage time of *T. asperellum* biomass. (**A**)—*T. asperellum* fresh biomass in 200× magnification. (**B**)—*T. asperellum* biomass after a storage period of two weeks or more in 400× magnification. (**C**)—Hyphae with chlamydospores in fresh biomass. (**D**)—Hyphae with conidiophores, phialides and conidia after a storage period of two weeks or more. Biomass storage time: (**C**,**E**,**G**,**I**)—0 days; (**D**,**F**,**H**,**J**)—two weeks. Antagonistic effect: (**E**,**F**)—after 3 days; (**G**,**H**)—after 5 days ((**G**)—on a black background); (**I**,**J**)—after 7 days.

*T. asperellum* developed mycelium and chlamydospores during submerged fermentation (Figure 1A). Chlamydospores had formed at the hyphal tips as well as intercalary within hyphae. The resulting biomass was colourless (Figure 1C), viable (Table 2) and showed antifungal activity against *F. graminearum* that did not appear immediately or after three days (Figure 1E) but was observed after five and seven days of co-cultivation in Petri plates (Figure 1G,I). After two weeks of storage in Petri dishes, pigmentation began, and the *Trichoderma* biomass turned green (Figure 1D). Microscopy showed hyphae with conidiophores and conidia (Figure 1B). 

In all treatments, *F. graminearum* was overgrown with *T. asperellum* in seven days, and accordingly, the larger the area occupied by *T. asperellum* the smaller the area colonized by *F. graminearum*, as shown in Figure 1. The antifungal activity of *T. asperellum* persisted throughout the experiment for at least eight weeks.

## 4. Discussion

*Trichoderma* submerged fermentation yields unpigmented biomass, usually without producing conidia [12] or substances whose biosynthesis involves the differentiation of hyphae in the atmosphere. Conidia have dispersing and resting functions in nature [28] and show a better long-term viability (an important feature for microbial preparations) than hyphae. The present experiments showed that posttreatment procedures (Table 1) of the obtained biomass had the capacity to induce conidia formation in *T. asperellum* mycelium. Biomass treatment methods were chosen to slow fungal metabolism while promoting conidiation. As reported in [7], the acidification of biomass to pH 4 and the addition of up to 10% additional nutrient starch to *Trichoderma* biomass improved its viability. Various species of the genus *Trichoderma* are known to be able to maintain viability in the range of pH 2 to pH 8 [29]. In several species of *Trichoderma*, low pH seems to be a determinant for the formation of conidia [30]. This study on *T. asperellum* MSCL 309 confirmed this fact.

Several heavy metal ions at low concentrations are required for fungal growth, but at higher concentrations, they can be toxic, completely inhibiting the development of microorganisms [31]. CuSO_4_, when added at low concentrations to biomass, acts as a metabolic inhibitor, inhibiting fungal growth and thus increasing the storage time of biomass [7]. Our results show that the addition of CuSO_4_ in combinations with HCl and starch produced high values of colonization, but they were not higher than those achieved by the application of HCl alone (Table 2). Considering the environmental toxicity of CuSO_4_ [32], we recommend using hydrochloric acid as a treatment option.

In addition to dissolved chemicals, air is also an important factor in mycelial differentiation, and in our experiments, air directly affected the mycelium during drying in Petri plates [33]. Oxygen is important for maintaining cell viability and metabolism, including antifungal activity. Another study [34] examined the storage time of *T. viride* by adding talc and charcoal to biomass. Samples with higher concentrations of talc retained greater viability than those with lower concentrations of talc, but preparations with added charcoal showed the opposite effect. Posttreatment of *T. asperellum* MSCL 309 with talc could also be investigated in the future.

With changes in the environment and stress conditions, we can probably explain the decrease in the surface area colonized by *Trichoderma* (Table 2) observed in the first week after the removal of biomass from the bioreactor. This biomass (Figure 1C) consisted of hyphae with chlamydospores (Figure 1A), but hyphae are known to grow at their tips; Ref. [35] described and analyzed in detail the morphogenesis of fungi under stressful conditions and its detailed cellular and molecular mechanisms, including the development of conidiophores at the air interface. It takes several days for conidia to form. In several species of *Trichoderma*, mechanical damage also triggers the production of conidia [36]. Research using gene knockout and complementation [37] identified the *vel1* gene as a key regulator of morphogenetic traits (conidiogenesis, etc.), secondary metabolism (including antibiosis and pigmentation) and biocontrol (including mycoparasitism).

The study [38] showed that *T. asperellum* biomass, as long as it contains living cells, has antifungal activity, as demonstrated in in vitro dual culture experiments with the Fusarium head blight pathogen *F. graminearum* (Figure 1). Thus, the antifungal activity of *T. asperellum* depended on the viability of the biomass as measured by its colonized surface area, and acidification of the biomass with HCl (treatment No. 2) increased its antifungal activity compared to the untreated biomass.

*Trichoderma* inhibited the growth of the pathogenic mycelium. The fungal pathogen *Fusarium graminearum* is the most common causal agent of Fusarium head blight (FHB) in many parts of the world. This destructive disease, commonly but perhaps inappropriately known as scab, affects wheat, barley and other small grains both in temperate and in semitropical areas [39]. Diseases caused by *F. graminearum* are of particular concern because harvested grains frequently are contaminated with harmful mycotoxins such as deoxynivalenol (DON) [40]. DON is a mycotoxin produced by the plant pathogenic fungi *F. graminearum* and *F. culmorum*. Other mycotoxins produced by FHB-causing fungi include nivalenol, T-2 toxin, and zearalenone. Ingestion of mycotoxin-contaminated food and feed can lead to toxicosis in humans and animals, respectively [41].

According to published information, the *T. asperellum* strain MSCL 309 also inhibits the growth of the conifer root and butt rot fungi *Heterobasidion annosum* s.s. and *H. parviporum* [25].

## 5. Conclusions

All the posttreatment methods studied improved the viability of *Trichoderma*, at least in terms of the area of the colonized surface. Four of the five relatively viable treatments contained heavy metal copper (II) sulphate pentahydrate at 20 or 60 mg/L. To avoid contamination of environmentally friendly *Trichoderma* preparations with Cu, in the future, we recommend only biomass acidification with HCl. Results of HCl application did not differ significantly from the results of CuSO_4_ application. There was no synergy or antagonism between the addition of HCl and Cu sulphate. Treatment with starch, alone or in combination with CuSO_4_ and/or HCl, did not significantly affect fungal viability compared to the control.

In the future, we recommend the use of acidification of *T. asperellum* biomass obtained by submerged fermentation. Other parameters for fermentation and posttreatment procedures should be studied in more detail to obtain long-term viable *T. asperellum* preparations.

## Figures and Tables

**Table 1:** Biomass treatments.

Treatment	1 M HCL	CuSO_4_×5H_2_O 2 mg/mL (Final Concentration, µg/mL)	Starch
1	-	-	-
2	X	-	-
3	-	X (20)	-
4	-	X (60)	-
5	X	X (20)	-
6	X	X (60)	-
7	-	-	X
8	X	-	X
9	-	X (20)	X
10	-	X (60)	X
11	X	X (20)	X
12	X	X (60)	X

**Table 2:** Surface colonization with *T. asperellum* as a function of biomass storage time. Treatment of biomass: 1—control; 2—HCl; 3—CuSO_4_×5H_2_O 20 mg/L; 4—CuSO_4_×5H_2_O 60 mg/L; 5—HCl and CuSO_4_×5H_2_O 20 mg/L; 6—HCl and CuSO_4_×5H_2_O 60 mg/L; 7—starch; 8—HCl and starch; 9—CuSO_4_×5H_2_O 20 mg/L and starch; 10—CuSO_4_×5H_2_O 60 mg/L and starch; 11—HCl, CuSO_4_×5H_2_O 20 mg/L and starch; 12—HCl, CuSO_4_×5H_2_O 60 mg/L and starch. The standard deviations were calculated from three repetitions. A significant difference was established for all rows (*p* < 0.05). Significantly different mean values in the same column are indicated by different superscripts (a–e) (Duncan; *p* < 0.05).

Treatment	Week					
	0	1	2	4	6	8
1	27.28 ± 0.74 ^c^	21.77 ± 0.00 ^b^	30.86 ± 0.00 ^b^	64.43 ± 6.78 ^b,c,d^	69.44 ± 1.18 ^b,c^	52.16 ± 1.02 ^e^
2	32.12 ± 0.80 ^a^	19.80 ± 1.25 ^c^	34.68 ± 0.83 ^a,b^	65.79 ± 1.14 ^c^	83.04 ± 2.57 ^a^	65.89 ± 3.43 ^a,b^
3	28.49 ± 1.50 ^c^	22.93 ± 2.02 ^a,b^	33.42 ± 1.63 ^a^	63.98 ± 0.00 ^d^	73.29 ± 3.62 ^b,c^	64.03 ± 2.26 ^a,b^
4	30.86 ± 0.00 ^b^	21.97 ± 2.63 ^b,c^	34.78 ± 2.49 ^a,b^	75.54 ± 7.34 ^a^	80.99 ± 1.27 ^a^	62.23 ± 1.11 ^c^
5	30.86 ± 1.50 ^b^	19.95 ± 2.51 ^b,c^	33.42 ± 1.63 ^a,b^	77.14 ± 3.72 ^a^	75.54 ± 7.34 ^a,b,c^	63.98 ± 0.00 ^b^
6	30.86 ± 0.00 ^b^	18.79 ± 0.61 ^c^	30.86 ± 0.00 ^b^	68.03 ± 6.97 ^a,b^	73.49 ± 6.04 ^a,b,c^	63.22 ± 10.03 ^a,b,c^
7	30.86 ± 0.00 ^b^	21.97 ± 2.63 ^b,c^	33.57 ± 3.26 ^a,b^	66.77 ± 10.31 ^a,b^	67.64 ± 2.32 ^c^	58.78 ± 1.00 ^d^
8	30.86 ± 0.00 ^b^	17.87 ± 1.19 ^b,c^	28.49 ± 1.50 ^c^	62.53 ± 5.57 ^c^	71.34 ± 2.38 ^b,c^	63.98 ± 0.00 ^b^
9	30.86 ± 0.00 ^b^	26.31 ± 2.88 ^a,b^	35.99 ± 0.00 ^a,b^	75.29 ± 4.89 ^a^	71.34 ± 2.38 ^b,c^	54.21 ± 6.21 ^d,e^
10	30.86 ± 0.00 ^b^	26.17 ± 1.44 ^a^	41.10 ± 8.05 ^a,b^	60.53 ± 2.19 ^d^	71.34 ± 2.38 ^b,c^	67.64 ± 2.32 ^a^
11	30.86 ± 0.00 ^b^	25.01 ± 0.70 ^a^	39.94 ± 8.79 ^a,b^	69.44 ± 1.18 ^b^	73.29 ± 3.62 ^b,c^	58.88 ± 3.24 ^b,c^
12	30.80 ± 1.50 ^b,c^	17.87 ± 1.19 ^b,c^	40.21 ± 2.68 ^a,b^	67.64 ± 2.32 ^c^	71.29 ± 0.00 ^b^	71.49 ± 4.77 ^a^

## Data Availability

All data generated or analyzed during this study are included in this article.
